# Molecular and Serological Prevalence of HCMV in Iranian Patients with Breast Cancer 

**DOI:** 10.31557/APJCP.2021.22.7.2011

**Published:** 2021-07

**Authors:** Mohsen Nakhaie, Javad Charostad, Azarakhsh Azaran, Seyyed Ali Mohammad Arabzadeh, Azim Motamedfar, Sara Iranparast, Fatemeh Ahmadpour, Abdolhasan Talaeizadeh, Manoochehr Makvandi

**Affiliations:** 1 *Cancer Research Center, Ahvaz Jundishapur University of Medical Sciences, Ahvaz, Iran. *; 2 *Department of Medical Virology School of Medicine, Ahvaz Jundishapur University of Medical Sciences, Ahvaz, Iran. *; 3 *Department of Medical Microbiology, Kerman University of Medical Sciences, Kerman, Iran. *; 4 *Department of Nuclear Medicine, School of Medicine, Golestan Hospital, Ahvaz Jundishapur University of Medical sciences, Ahvaz, Iran. *; 5 *Department of Immunology, Ahvaz Jundishapur University of Medical Sciences, Ahvaz, Iran. *; 6 *Department of Clinical Biochemistry, Faculty of Medicine, Ahvaz Jundishapur University of Medical Sciences, Ahvaz, Iran. *

**Keywords:** Cytomegalovirus, prevalence, breast neoplasms, Iran

## Abstract

**Background:**

Human cytomegalovirus (HCMV) is prevalent viral infection involved in several human cancers including breast cancer. The presence of HCMV genome in breast cancer tissue and footprint of viral last exposure patient’s serum are considered as important factor in the process of breast cancer development.

**Objectives:**

This study aimed to investigate molecular and serological epidemiology of HCMV in patients with breast cancer in Iran for first time.

**Methods:**

In our case-control study, 98 samples of breast tissue, including 49 cancerous (case) and 49 adjacent non-cancerous tissue were collected (control). In addition, we collected sera samples from all patients (n=49) and healthy individual (n=49). Seroprevalence of HCMV was assessed by Enzyme-linked immunosorbent assay (ELISA) and detection of HCMV genome was performed using Nested-PCR method.

**Results:**

HCMV genome found in 16.3% (8/49) of cases tissue and 2% (1/49) of controls tissue. In patients group, the levels of anti-CMV IgG and IgM were 93.9% and 2% compared to 69.4% and 4.1% in healthy individuals, respectively. There was a statistically difference between the anti-CMV IgG in patients and healthy control (p= 0.002). We found 75% of (6/8) HCMV genome positive PCR samples were also positive for their anti-CMV IgG in cases which was statistically significant (p= 0.01).

**Conclusions:**

Our result showed significant presence of HCMV genome and anti-CMV IgG in patients, supporting the role of HCMV in breast cancer.

## Introduction

Breast cancer is the most common invasive cancer and the second leading cause of cancer death in females after lung cancer (Bray et al., 2018). Only in 2018, 2,088,849 and 626,679 new cancer cases and related death, respectively, occurred in worldwide (Bray et al., 2018). Histologically, most of breast cancer cases start from milk ducts and called ductal carcinoma. The second most frequent type of breast cancer, lobular carcinoma, begins in the milk-producing glands of the breast that is histologic forms of this malignancy (Li et al., 2005; Sharma et al., 2010). Less-common other types of breast cancer include tubular, mucinous, comedo, and medullary type(Li et al., 2005). Tumor grades include types I, II and III, which are used to describe the rate of growth and spread of breast cancer cells (Rakha et al., 2010). Breast cancer is a multifactorial disorder which the factors such as genetic, hormone replacement therapy, ethnicity, age, sex, abortion, alcohol, obesity and nulliparity may be involved in its pathogenesis (Martin and Weber, 2000; Momenimovahed and Salehiniya, 2019). 

Among the external factors, biological carcinogens are demonstrated to play a key role in the development of human cancers (Parsa, 2012; Alibek et al., 2013). According to the reports of the International Agency for Research on Cancer (IARC), the biological carcinogens contribute in 18-20% of cancers which viruses comprise the major category of these elements (Alibek et al., 2013). In the pathogenesis of breast cancer, viruses are thought to be influential players with ability of affecting substantial biological process (Alibek et al., 2013). In this regard, the role of Human cytomegalovirus (HCMV) is highlighted (Geisler et al., 2019). 

Although the role of these viruses in the breast cancer are controversial, but some studies reported an association between HCMV and breast cancer (Geisler et al., 2019).

HCMV is a β-herpesvirus from Herpesviridae family, also known as human herpes Virus 5 (HHV-5), that carried by 50% to 100% of the adult population in the world (Michaelis et al., 2009; Al Nuimi et al., 2018). Encoding proteins with oncogenic functions such as US28, the ability of cellular transformation “in vitro”, oncomodulation features and manipulation of the innate and adaptive immune responses have led to HCMV being considered as a virus with tumorigenic properties (Nakhaie et al., 2020). Investigations report HCMV associates with different cancers such as breast, colon, prostate, glioblastoma, medulloblastoma and neuroblastoma (Nauclér et al., 2019). The breast epithelium tissue is known as potential reservoir for persistent HCMV infection in humans (Harkins et al., 2010a). The evidence suggests the presence of HCMV in breast carcinoma and various reports have detected virus in most early and advanced stages of breast cancers (Geisler et al., 2019). Conversely in most of cases, HCMV is detected in a low range or even not found in normal tissue (Geisler et al., 2019).

It is worth noting that incidence of breast cancer follows the certain geographical patterns which is not explainable with differences in known risk factors between the nations (Sung etal., 2021). Interestingly, serologic data propose viral infections such as HCMV are among these variable factors related to special geographical patterns. It is believed that late exposure to HCMV as a common virus in certain populations may promote the risk of breast cancer (Richardson, 1997). The investigations refer to the evidence showing frequency of breast cancer is higher in populations where exposure to HCMV may happen late than in populations where almost individuals are exposed in childhood (Richardson, 1997). In this line, researchers have pointed to increased value of anti-CMV IgG antibodies in breast cancer patients as against normal individuals (El Shazly et al., 2018). Seroprevalence of HCMV in the adult population has been estimated from 45% to 100%, depending on various factors such as age, geographical area, social and economic status(Cannon et al., 2010). 

Therefore, in the case of HCMV which its prevalence follows certain geographical patterns, only exploring of viral genome in breast cancer tissue seems to be inadequate. 

In the present study for the first time in Iran we investigated the association between HCMV infection and the development of breast cancer via assessment of the presence of HCMV genome in cancerous (case) and adjacent non cancerous tissue (control), as well as measuring the presence of IgM and IgG antibodies in patient’s serum samples.

## Materials and Methods

*Study population and collection of the specimens*

49 pairs of cancerous tissue and normal tissue adjacent to cancerous samples were collected at different hospitals in Ahvaz province during April 2020 and October 2020. The new cases with confirmed pathological evidence were included and the patients who had a cancer history, metastatic cancer and receiving neoadjuvant chemotherapy were excluded. In all women diagnosed with breast cancer, a breast examination was performed by an experienced surgeon. In addition, tissue samples were evaluated for the histopathologic assessment of breast cancer according to the WHO criteria by two experienced pathologists to ensure the correct diagnosis. All tissue samples were immediately snap frozen in liquid nitrogen after biopsy and stored at -80°C. 

In addition to tissue specimens, 49 blood samples from all enrolled patients and 49 blood samples from healthy women (with no history of malignancy) as a control group were collected, and the serums were taken and stored at -80°C until analysis. 

The study was approved by ethics committees of Ahvaz Jundishapur University of Medical Sciences, Ahvaz, Iran under the ethics code of IR.AJUMS.REC.1399.618. Also, all patients signed an informed consent form concerning the participation in the study.

*Genomic DNA preparation and Polymerase Chain Reaction*

**Figure 1:**
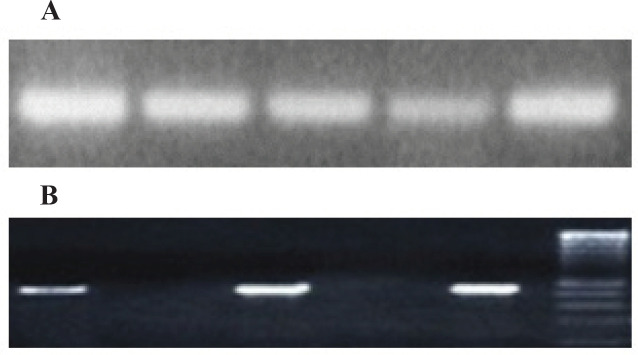
Positive Polymerase Chain Reaction Bands Corresponding β-Globin (A) and CMV- DNA Polymerase (B)

DNA was extracted from 25-30 mg fresh tissue (Case and control) using QIAamp® DNA Mini kit ) QIAGEN, Germany, Cat No./ID: 51304) according to the manufacturer’s instructions. The extracted DNA was stored at -70°c until PCR amplification. All extracted DNA samples were initially subjected to PCR using PCO3/PCO4 primers to evaluate amplification of human β-globin gene (as internal control) and confirm the quality of the extracted DNA as previously described (Charostad et al., 2020). The primer sequences were as follows: PCO3:5 ´ ACACAACTGTGTTCACTAGC-3’/PCO4: 5’ CAACTTCATCCACGTTCACC-3’ with fragment size of 110 bp. All beta globin positive samples underwent further investigation (Figure 1). Nested PCR method was employed for screening the presence of CMV in all Specimens. The DNA polymerase coding region was amplified by nested PCR, serving the following outer oligonucleotide primers: 5’GTCGTGTTTGACTTTGCCAGC -3’and 5’GTCTTGCGCACCAGATCCAC-3’ (748bp), and inner oligonucleotide primers: 

5’ GCATCATCCTGGCTCACAACC -3’ and 5’ GTCCGTGTCCCCGTAGATG -3’ (499 bp). Both PCRs were performed with the final volume of 25μl containing of, 1X PCR buffer, 100 μM dNTPs, 1.5 mM MgCl_2_, 1 units of Taq polymerase enzyme, 0.5μl of each outer and inner primers (25 pmol) and 300 ng of DNA extracted/product. The program of PCR cycling was carried out along these lines: 1) 10 min at 94°C, 2) 45 s at 94°C, 45 s at 51°C and 45 s at 72°C for 35 cycles; and 3) 5 min at 72°C in first PCR and 1) 10 min at 94 °C, 2) 30 s at 94°C, 45 s at 49°C and 30 s at 72°C for 35 cycles; and 3) 5 min at 72°C in second PCR. All PCR products were loaded in 1.5 % agarose gel and visualized with UV (Figure 1).

*Enzyme Linked Immunosorbent assay (ELISA)*

We tested serum samples for anti-CMV IgG and IgM by using a commercial microparticle enzyme immunoassay (Equipar, Italy) in accordance with the manufacturer’s instructions. Optical Density (OD) of all samples is read by ELISA reader (Epoch, USA). The cut-off value 0.5 U/ml is considered for determination positive and negative results. 

*Statistical analysis*

Data were analyzed using Statistical Package for the Social Sciences (SPSS) version 22 IBM and GraphPad Prism, version 8.0.2 (GraphPad Software, Inc.). The relation of groups with viruses and their association with clinical pathological factors of patients was determined using the Chi-square test or the non-parametric Fisher’s exact test. Probability (p-value) equal or less than 0.05 was considered statistically significant.

## Results

*Patients’ characteristics*

The clinical data of the patients are illustrated in Table 1. The patients age ranged from 24 to 68 years in the cancerous group (mean 47.4 ± 12.7 years) and 27 to 64 in healthy individuals (mean 45.3 ± 14.7 years). 

Of 49 patients with breast tumors: 25 (51%) were in the right breast, 22 (44.8%) were in the left and 2 (4.1%) were Bilateral breast cancer. Histological analysis showed 42 (85.7%) samples were invasive ductal carcinoma, 4 (8.1%) invasive lobular carcinoma, 2 (4.1%) invasive medullary carcinoma and 1 (2%) were invasive tubular carcinoma (Table 1). 

*Screening for HCMV genome in breast cancer tissue*

**Figure 2:**
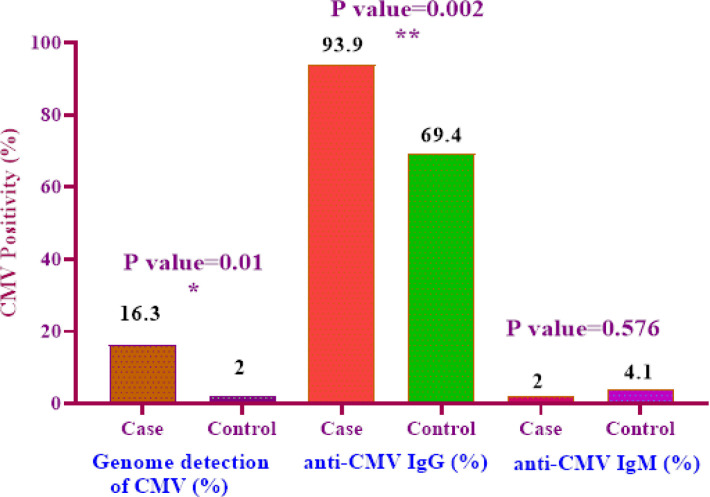
Percentage of CMV PCR Positivity, Anti-CMV IgG and Anti-CMV IgM in Patients and Healthy Groups

CMV DNA was detected in 8 out of 49 cases (16.3%) and 1 (2%) out of 49 controls (Figure 2). The detection of CMV DNA in cancerous (case) rather than adjacent non-cancerous tissue (control) was significant (Table 1; p= 0.01). The frequency of virus in grades I, II and III were 4, 1 and 3, respectively. Investigation on the association between the HCMV and the type of cancer showed that most of the positive cases of the HCMV (n=7) were detected in the invasive Ductal carcinoma (Table 1).

*Screening for HCMV antibody in breast cancer and normal control serum*

Evaluation of HCMV antibody levels was determined by ELISA in breast cancer patients. 46 (93.9 %) samples from the patients group and 34 (69.4 %) samples from the healthy group were positive for anti-CMV IgG and 1 (2%) and 2 (4.1%) samples were positive for anti-CMV IgM in case and control group, respectively (Figure 2). The difference was statistically significant in the index value (IV) of anti-CMV IgG antibodies between the patients group and the healthy group (Figure 2; p= 0.002). On the other hand, no statistically difference between clinical features in reference to HCMV antibodies status was observed.

*Correlation between HCMV antibodies and presence of viral genome*

According to 8 PCR HCMV positive cases in breast tissue, 6 (75%) samples were positive for their anti-CMV IgG which was statistically significant (Figure 2; p= 0.01). We also, observed no significant result between anti-CMV IgM positivity and genome presence of HCMV in corresponding tissue.

**Table 1:** Patients Clinical Characteristics According to Presence Viral Infection

Parameter	Total	CMV+	CMV-
Age (mean±sd)	47.4 ± 12.1	46.5 ± 14.1	47.7 ± 11.8
Type of cancer,n (%)			
Ductal	42 (85.7)	7 (16.7)	35 (83.3)
Lobular	4 (8.2)	1 (25)	3 (75)
Medullary	2 (4.1)	0	2 (100)
Tubular	1 (2)	0	1 (100)
Grade, n (%)			
I	20 (40.8)	4 (20)	16 (80)
II	12 (24.5)	1 (8.3)	11 (91.7)
III	17 (34.7)	3 (17.6)	14 (82.4)
Metastasis, n (%)	8 (100)	3 (37.5)	5 (62.5)

## Discussion

HCMV, a human beta-herpes virus, belongs to the herpesviruses family (Michaelis et al., 2009). Virus ability to transform cells in vitro and its association with several cancers have been described (Nauclér et al., 2019). Breast tissue is considered as potential reservoirs for HCMV and its identification in breast cancer tissue is suggested (Harkins et al., 2010). However, there are conflicting data that report different detection rate of HCMV in breast cancer. For example, a conducted study by Bakhtiyrizadeh et al., and Mohammadizadeh et al., showed none of the breast cancer and non-cancerous samples were positive for HCMV genome (Bakhtiyrizadeh et al., 2017; Mohammadizadeh and Mahmudi, 2017). In contrast, another study by karimi et al., (2016) found presence of HCMV genome in 58% (26/50) of breast cancer samples. In the present study, in first step we investigated the presence of HCMV genome in 49 breast cancer tissue and their adjacent normal tissue (n=49) using nested-PCR.

We found HCMV genome in 9.2 % (9/98) all specimens. Our data indicate a higher frequency of HCMV in the tumor tissue (16.3 %) rather than normal group (2 %) which was statistically significant (Figure 2; p= 0.01). In agreement with us, several studies from different geographical areas showed higher frequency of virus in breast cancer rather than non-cancerous samples (El Shazly et al., 2018; Sepahvand et al., 2019).

It has been hypothesized that absence or low detection of HCMV genome in breast cancer tissue cannot reject the influential role of HCMV in development of breast cancer (Richardson et al., 2015). In this line, delayed exposure to HCMV has been displayed to increase risk of breast cancer (Yasui et al., 2001).Therefore, serologic assessments may lead to better understanding of the exact viral etiology in this process.

The seroprevalence of HCMV in different regions of world depends on various conditions such as socioeconomic status (Cannon et al., 2010; Zuhair et al., 2019). HCMV possess the highest prevalence in Africa and Asia and the lowest prevalence is reported from in European countries and the United States (Cannon et al., 2010). According to a recently conducted study, the pooled prevalence rate of HCMV IgM and IgG among the Iranian women 0.06% and 90% was estimated, respectively (Sharghi et al., 2019).

To our knowledge, there is no report from Iran concerning the prevalence rate of anti-CMV Abs in serum of patients with breast carcinoma. However, the correlation between high HCMV seroprevalence and female’s disease has been suggested. For example, a conducted study by Jahromi et al., (2010) in Iran found that there is a statistical connection between high HCMV seroprevalence and increased risk of abortion.

In this investigation, the level of anti-CMV IgG was higher in patient’s serum than the healthy group (Figure 2). In patients group, the levels of anti-CMV IgG and IgM were 93.9% and 2% compared to 69.4% and 4.1% in healthy individuals, respectively (Figure 2). There was a statistically difference between the anti-CMV IgG in patient and healthy controls (Figure 2; p= 0.002). In addition, statistically difference between the presence of the virus in the tissue and its anti-CMV IgG was observed (Figure 2; p= 0.01). We found 6 (75%) HCMV genome positive PCR samples in cases were also positive for their anti-CMV IgG which was statistically significant (Figure 2; p= 0.01).

In the case of breast cancer, several investigations have proposed high prevalence rate of anti-CMV IgG that shows the history of viral late exposure in the patients. (Cox et al., 2010; Richardson et al., 2015). A conducted study in breast cancer patients from Iraq by Nuimi et al., showed levels of anti-CMV IgG and IgM were 100% and 8.3%, respectively (Al Nuimi et al., 2018). Additionally, in another study conducted in Egypt in 2017, anti-CMV IgG found in all cases (100%), in contrast anti-CMV IgM was not detected (0%) (El Shazly et al., 2018). Performed investigations from New Zealand and Australia have reported lower of anti-CMV IgG levels in breast cancer with prevalence rate of 70% and 59%, respectively (Richardson et al., 2004; Richardson et al., 2015b). 

With respect to Anti-CMV IgM, it should be noted that IgM is generated following initial infection, and as well as due to re-infection of HCMV, hence, probably explaining its low positivity.

In Conclusion, Our result showed significant presence of HCMV genome and anti-CMV IgG in breast cancer patients. Similar to previous investigations, our study introduces last exposure to HCMV as a probable factor that proceeds the development of breast cancer. The data may suggest importance of combination of molecular and serologic assessments as a useful tool for better understanding of HCMV contribution in disease. In addition, the current study may emphasize on anti-CMV vaccinations as preventive strategy for HCMV-related disease. 

## Author Contribution Statement

Study concept and design: Manoochehr Makvandi, Mohsen Nakhaie, and Azarakhsh Azaran; Analysis and interpretation of data: Mohsen Nakhaie, Javad Charostad, and Azim Motamedfar; Drafting of the manuscript: Javad Charostad; Critical revision of the manuscript for important intellectual content: Seyyed Ali Mohammad Arabzadeh; Statistical analysis: Sara Iranparast, and Fatemeh Ahmadpour.
